# Evaluating the Effectiveness of Chatbot-Based Patient Education Compared to Traditional Patient Information Leaflets Related to Pediatric Anesthesia: A Pilot Cross-Sectional Study

**DOI:** 10.7759/cureus.108426

**Published:** 2026-05-07

**Authors:** Uday Ambi, Mahima Gajanan Kamath, Sneha Jossie, Salman M Soomar

**Affiliations:** 1 Anesthesiology, King's College Hospital, Dubai, ARE; 2 Quality and Research, King's College Hospital, Dubai, ARE

**Keywords:** artificial intelligence and education, chatgpt, large language models (llms), medical communication, parental satisfaction, patient information leaflets, pediatric anaesthesia, readability score

## Abstract

Background

Clear, accurate, and empathetic communication is essential in pediatric anesthesia, where parental anxiety and information needs are high. Traditional patient information leaflets (PILs), while clinically robust, may lack emotional engagement. Large language model (LLM)-based chatbots, such as ChatGPT and Google Gemini, offer a novel, interactive approach to patient education, yet their role in pediatric anesthesia remains inadequately explored.

Objective

To evaluate and compare the readability, accuracy, completeness, sentiment, and parental satisfaction of artificial intelligence (AI)-generated patient education materials (ChatGPT and Google Gemini) with a clinician-authored departmental PIL (DPIL) for pediatric general anesthesia.

Methods

This pilot cross-sectional study evaluated responses generated by ChatGPT and Google Gemini to seven frequently asked questions derived from the departmental PIL. Three blinded leaflets were presented in randomized order using a computer-generated sequence and evaluated by 10 anesthetists for accuracy and completeness using 10-point Likert scales. Readability was assessed using Flesch Reading Ease and Flesch-Kincaid Grade Level scores. Sentiment analysis and parental satisfaction were also assessed. Both descriptive and inferential statistical analyses were performed.

Results

The DPIL demonstrated the highest readability, followed by ChatGPT, with Gemini scoring the lowest. All materials exceeded the recommended sixth-grade readability level. No significant differences were observed in accuracy or completeness among the three sources (p > 0.05). Parents consistently perceived ChatGPT responses as more reassuring and relatable, while the DPIL was viewed as informative but formal. Gemini responses were often considered linguistically complex. ChatGPT demonstrated a neutral and more empathetic sentiment compared with the other leaflets.

Conclusion

Clinician-authored PILS remain the most reliable source of pediatric anesthesia information. AI-generated content, particularly ChatGPT, may enhance clarity and emotional reassurance when used as a clinician-reviewed adjunct rather than a replacement.

## Introduction

Large language models (LLMs), a subset of artificial intelligence (AI) and linguistics, are designed to help computers understand and analyze human language [1]. These sophisticated algorithms leverage deep learning techniques and extensive datasets to comprehend and generate information in a manner that closely emulates human communication [2]. Their advanced natural language processing (NLP) capabilities have led to a recognition of their potential enhanced analytic capacity across various fields, including medicine and dentistry [1,3]. Patient education and counseling represent a particularly promising domain for LLM application, enabling them to generate human-like and accessible responses.

Anesthetists often face time constraints that limit their ability to provide comprehensive patient education, making LLMs a potential solution for efficient information delivery [2].

The emergence of AI chatbots, such as ChatGPT (Chat Generative Pre-Trained Transformer, version 4.0) and Google Gemini (formerly Google Bard, version 2.5), presents a promising avenue for patient education, offering dynamic and interactive responses to patient inquiries [4]. These tools can distill complex medical concepts into accessible, layman-friendly language, thereby enhancing comprehension and engagement [5].

Despite their potential, concerns regarding the reliability, accuracy, and readability of LLM-generated content persist [4]. A significant limitation across all models is the consistent lack of source citations and transparency regarding their training data, which can include inaccuracies, outdated details, or biases, leading to "hallucinations"-plausible but incorrect responses. This raises a critical question regarding whether LLMs should prioritize detailed accuracy or simplicity for patient understanding [2].

Comparative analyses of ChatGPT and Google Gemini in patient education have yielded mixed results. A comparison of ChatGPT-4o, Claude 3, and Gemini for anesthetic and perioperative care patient education found Gemini to be most reliable, followed by Claude 3, but noted that ChatGPT-4o was most comprehensible despite lacking clinical depth and citations [2]. Another review comparing ChatGPT and Gemini in medical inquiries noted that ChatGPT generally demonstrated higher accuracy and shorter responses across various specialties, but Gemini outperformed ChatGPT in emergency scenarios and specific renal diet contexts, suggesting varied strengths [6]. In generating patient information leaflets for laparoscopic hernioplasty, ChatGPT and Gemini were comparably accurate, but ChatGPT showed significantly better completeness than Meta AI, with all models producing text above the recommended sixth-grade reading level [7].

Crucially, while these studies explore LLM utility in general medical or adult surgical contexts, their application in pediatric anesthesia patient education remains significantly underexplored. Pediatric patients and their guardians have unique communication needs, often requiring particularly clear, empathetic, and age-appropriate explanations to mitigate anxiety and ensure informed decision-making for vulnerable populations. Given the current limitations of LLMs, such as inconsistent accuracy, the potential for hallucinations, and the absence of a "human touch", rigorous evaluation is essential. Therefore, a comparative analysis of ChatGPT and Google Gemini in generating patient education responses specific to pediatric anesthesia is warranted to assess their respective strengths and weaknesses in this specialized and sensitive area, ultimately informing their responsible integration to support current clinical practices and enhance patient safety.

Aims/objectives

The primary objective of this study is to evaluate how effectively three different patient information leaflets communicate information about pediatric general anesthesia to parents and anesthesiologists. The secondary objectives are to compare the overall satisfaction scores given by parents for each leaflet, with particular emphasis on emotional tone, clarity, and reassurance; to compare anesthesiologists' assessments of ease of information delivery and comprehensibility using a 1-10 scale; and to determine which leaflet (ChatGPT, Gemini, or KCH) is perceived as most effective overall by each group.

## Materials and methods

Google Gemini and ChatGPT were tasked with generating responses to seven frequently asked questions (FAQs) sourced from the Patient Information Leaflet (DPIL) designed and endorsed by the Department of Anaesthesia.

The exact prompt used for both AI models was as follows: "Provide parent friendly answers to the following frequently asked questions regarding pediatric general anesthesia." Responses were generated using ChatGPT (GPT-4 series, OpenAI, San Francisco, CA) and Google Gemini (version 2.5; Google DeepMind/Alphabet Inc., Mountain View, CA) in May 2025. A single response was generated for each question without regeneration or iterative prompting to reflect typical real-world use. Default model settings were applied, without using the PRO version. The corresponding outputs have been included in the supplementary material to ensure transparency and reproducibility.

The study was designed as a pilot cross-sectional comparative analysis; therefore, a formal a priori sample size calculation based on hypothesis testing was not undertaken. Instead, the sample size was determined pragmatically to evaluate feasibility, estimate variability, and generate preliminary effect size estimates for future studies.

For expert evaluation studies assessing qualitative attributes, such as accuracy and completeness, a panel size of 5-15 reviewers is considered adequate to ensure reliability and consistency of ratings [8]. Accordingly, 10 anesthetist faculty members were chosen to evaluate the accuracy and completeness of responses on a 10-point Likert scale (1-10). The faculty who were involved in the preparation of the patient information leaflets (PILs) were excluded from the study.

Similarly, 10 parents were recruited using convenience sampling and were asked to label each response as either Satisfied, Neutral, or Not satisfied based on their subjective perception. Pilot study guidelines recommend including 10-30 participants per group to estimate variability and inform the future design of adequately powered studies [9].

The departmental PIL (DPIL) was prepared based on the FAQ sources from the Royal College of Anaesthesia, UK (RCOA). There were three samples (ChatGPT, Gemini, and DPIL), which were blinded as Samples A, B, and C, respectively. Leaflets were presented to participants in random order using a computer-generated randomization sequence to minimize bias.

Responses were recorded for readability score, which was assessed using Flesch-Kincaid Grade Level (FKGL) and Flesch Reading Ease (FRE) scores. The score lets you know the approximate educational level a person will need to be able to read a particular text easily. The comprehensibility of a document will be indicated on the FRE score by a number between 0 and 100. Scores around 100 mean that the document is extremely easy to read, while scores around 0 mean that it is highly complex and difficult to understand. These readability metrics are primarily based on factors such as the average number of words per sentence, the number of complex words, and the average number of syllables per word (Appendix).

Sentiment analysis was conducted to determine the emotional tone of each response (positive, neutral, or negative), providing insights into the overall sentiment conveyed by the patient education material generated by each sample.

Statistical analysis

Mean and standard deviation (SD) were calculated for quantitative continuous variables. Both descriptive and inferential statistical analyses were performed. Differences in accuracy and completeness were analysed using one-way ANOVA, followed by post-hoc Tukey honestly significant difference (HSD) testing. A complete case analysis was performed with no missing data to see the overall comparison between the AI bots and human intelligence.

Ethical clearance

Ethical clearance was sought and obtained from the Institutional Ethics Committee prior to the commencement of the study, in accordance with the institutional policies and the principles of the Declaration of Helsinki. Participation was voluntary, and participants were informed of their rights to withdraw from the study at any stage without any consequences. All responses were anonymized prior to analysis, and no identifiable or sensitive personal health information was collected or stored. The study involved only the evaluation of patient education materials generated by publicly available AI language chatbots and an existing departmental leaflet, with minimal risk to participants. Data were securely stored and used solely for research purposes.

## Results

A total of three pediatric anesthesia-related PILs were evaluated by 10 anesthetists and 10 parents, each generated by ChatGPT, Gemini, and the DPIL. 

Readability analysis 

Across all seven FAQ topics, the DPIL demonstrated the highest relative readability (mean: 56.8 ± 11.4), followed by ChatGPT (45.5 ± 20.39), while Gemini produced the least readable content (35.7 ± 12.8). The DPIL corresponded to a 10th-12th-grade-level readability, while ChatGPT and Gemini corresponded to a more difficult college level (Table 1). However, all materials exceeded the recommended sixth-grade reading level, indicating limited accessibility.

**Table 1:** Readability scoring: DPIL vs Gemini vs ChatGPT *Total score: 100 DPIL: departmental patient information leaflet

Readability scores	DPIL	Gemini	ChatGPT
What is a general anesthetic?	47.2	33.2	41.7
What are the benefits of a general anesthetic?	70.8	19.4	54.7
Are there any alternatives to a general anesthetic?	56.8	38.8	56.8
What happens before a general anesthetic?	56.8	46	64.7
How is a general anesthetic given?	63.7	57	64.7
How will my anesthetist know that my child is asleep?	37.3	25.4	15
What complications can happen?	65.6	30.3	21
Overall Mean ± SD	56.8 ± 11.4	35.7 ± 12.8	45.5 ± 20.39

Gemini consistently displayed lower scores across nearly all questions, particularly in explaining complications (30.3) and defining general anesthesia (33.2). ChatGPT outperformed Gemini in most categories but showed lower readability in explaining how anesthetists know that a child is asleep (15). The DPIL had the highest readability for the majority of the topics, especially when outlining the benefits of general anesthesia (70.8) and explaining the complications (65.6)

Accuracy and completeness ratings

Anesthetists rated each leaflet on a 10-point Likert scale for accuracy and completeness. The DPIL was perceived as the most clinically accurate and complete. ANOVA results revealed no significant differences in accuracy scores (p = 0.252) and completeness scores (p = 0.096) across the three sources (Table 2). No significant differences were observed in the scores between any pair of sources in post hoc analysis (all p > 0.05).

**Table 2:** Rating for accuracy and completeness *Total score: 10; *p < 0.05: significant Likert scale interpretation: 1-3: Poor, 4-6: Fair, 7-8: Good, 9-10: Excellent DPIL: departmental patient information leaflet; HSD: honestly significant difference Post-Hoc Tukey honestly significant difference (HSD) analysis: ChatGPT vs Gemini (p = 0.685); ChatGPT vs DPIL (p = 0.240); Gemini vs DPIL (p = 0.128)

Rating	DPIL	Gemini	ChatGPT	P-value
Accuracy (Mean)	6.7 + 3.13	8.1 + 2.13	7.6 + 2.41	0.252
Completeness (Mean)	6.5	8.2	7.3	0.096

Sentiment analysis

Ten parents were asked to label each response as either Satisfied, Neutral, or Not satisfied based on their subjective perception. ChatGPT received the highest proportion of "satisfied" responses, followed by the DPIL. Feedback regarding the Gemini leaflet was mixed, with several parents noting difficulty understanding longer or more complex phrasing.

Overall, among anesthetists, sentiment analysis demonstrated a mean sentiment score of 0.6 + 0.51 for the Gemini leaflet and 0.6 + 0.51 for the DPIL, both corresponding to an overall negative sentiment tone, whereas ChatGPT demonstrated a neutral sentiment with a mean score of 0 + 0.94. Sentiment scores were interpreted using a scale, where -1 indicated negative sentiment, 0 neutral sentiment, and +1 positive sentiment. Sentiment analysis suggested that ChatGPT responses carried more reassuring and empathetic language compared to the other two leaflets (Table 3).

**Table 3:** Sentiment analysis -1: Negative, 0: Neutral, 1: Positive

Rating	Anesthetists	Gemini	ChatGPT
Sentiment Score (Mean)	0.6 + 0.51	0.6 + 0.51	0 + 0.94
Sentiment Tone	Negative	Negative	Neutral

## Discussion

Our study demonstrates that AI-generated PILs show considerable potential, particularly in emotional tone and accessibility, but currently serve as an adjunct rather than a stand-alone replacement for traditional PILs.

The DPIL achieved the highest readability scores and was consistently rated by anesthetists as the most accurate and comprehensive. However, no statistically significant differences were observed in accuracy or completeness between the three sources. Although Gemini demonstrated the highest mean accuracy score, this was not statistically significant, and therefore, no definitive superiority can be concluded. This is not unexpected as clinician-authored materials are developed using validated guidelines and expert consensus, minimising the risk of factual errors or omissions. In pediatric anesthesia, where accuracy and safety are of utmost importance, this reliability remains a key strength of traditional leaflets.

However, parent feedback highlighted an important limitation of conventional materials: they can sometimes feel formal and emotionally distant. In contrast, ChatGPT-generated responses were frequently described as clearer, conversational, and reassuring. This reflects the broader challenge in medical AI of balancing linguistic simplicity with clinical fidelity, which aligns with existing literature suggesting that ChatGPT excels in generating empathetic, patient-friendly language, even when clinical depth may be limited [2,4]. For parents facing the anxiety of their child undergoing anesthesia, this supportive tone may play a meaningful role in improving understanding and reducing stress.

Despite these advantages, ChatGPT responses occasionally lacked completeness or failed to fully address nuanced clinical concerns. Similar findings have been reported in previous studies, where AI-generated anesthesia content was found to be accessible but sometimes insufficiently detailed or inconsistently accurate [7,10]. This underlines a fundamental challenge in AI-generated PILs, which is achieving simplicity of language without compromising the depth or accuracy of information.

Google Gemini performed poorly in this audit, with the lowest readability scores across most FAQ domains. Sections addressing complications and intraoperative monitoring were particularly challenging to understand. These findings are consistent with comparative studies demonstrating that LLMs vary in performance depending on clinical content and task complexity [11]. This variability highlights the importance of careful selection of the LLM and the need for clinician oversight while deploying AI tools for patient education. However, at the time of the study, the Gemini 3.0 series was launched. The version used in this study is Gemini 2.5 series.

Importantly, most published studies evaluating AI-generated patient education have focused on adult populations or general medical contexts [5,12]. Pediatric anesthesia presents unique communication challenges, as information must be conveyed not only accurately but also compassionately and in a way that reassures caregivers into making decisions on behalf of their children. This study therefore adds valuable insight into an underexplored yet highly sensitive area of clinical communication.

A major strength of this study is the inclusion of both anesthetist and parent perspectives, allowing assessment of clinical accuracy alongside emotional tone and clarity. The use of objective readability metrics strengthens the evaluation even more. Blinding of the leaflets helped reduce reviewer bias.

However, the study has its own limitations. The sample size is relatively small with a single-center design. Convenience sampling may introduce selection bias. Additionally, the subjective nature of parent-report could also introduce response bias. Readability formulas do not fully capture true comprehension or emotional impact. AI outputs were also generated using a single prompt, which may limit reproducibility. Additionally, the rapid evolution of AI models means findings may change as newer versions are released [6].

The findings support a hybrid approach to patient education, combining the accuracy and reliability of clinician-authored materials with clarity and empathy demonstrated by AI-generated content. AI tools, especially ChatGPT, may be useful for drafting parent-friendly language or improving tone, but all outputs should undergo clinician review to ensure safety and completeness. Transparent documentation of AI use and robust verification processes are essential to mitigate risks associated with outdated information [1].

Future research involving larger multi-centre cohorts and quantitative sentiment analysis would help strengthen the overall evidence base. As AI tools continue to evolve, ongoing evaluation will be crucial to ensure responsible and effective integration into pediatric anesthesia practice.

## Conclusions

This study demonstrated that clinician-authored PILs continue to provide the highest levels of accuracy and readability for pediatric anesthesia. AI-generated content, especially ChatGPT, demonstrated strengths in tone and accessibility but showed variability in clinical completeness. Gemini produced lower readability scores and less consistent explanations across key domains. Overall, these findings support a hybrid model in which AI tools are best used as supportive adjuncts to clinician-reviewed materials rather than standalone replacements.

## Appendices

Frequently asked questions used as a prompt reference

The following questions from the departmental patient information leaflet were used to generate responses from ChatGPT and Google Gemini using the prompt: “Provide parent-friendly answers to the following frequently asked questions regarding pediatric general anesthesia.”

1. What is a general anesthetic?

2. What are the benefits of general anesthesia?

3. Are there any alternatives to a general anesthetic?

4. What happens before a general anesthetic?

5. How is a general anesthetic given?

6. How will my anesthetist know that my child is actually asleep?

7. What complications can happen?
